# Endogenous Auxin Content Contributes to Larger Size of Apple Fruit

**DOI:** 10.3389/fpls.2020.592540

**Published:** 2020-12-03

**Authors:** Haidong Bu, Wenquan Yu, Hui Yuan, Pengtao Yue, Yun Wei, Aide Wang

**Affiliations:** ^1^Key Laboratory of Fruit Postharvest Biology, College of Horticulture, Shenyang Agricultural University, Shenyang, China; ^2^Mudanjiang Branch of Heilongjiang Academy of Agricultural Sciences, Mudanjiang, China

**Keywords:** apple, fruit size, cell size, auxin, *MdTAR1*, *MdYUCCA6*

## Abstract

Fruit size is an important economic trait that is controlled by multiple genes. However, the regulatory mechanism for fruit size remains poorly understood. A bud sport variety of “Longfeng” (LF) apple (*Malus domestica*) was identified and named “Grand Longfeng” (GLF). The fruit size of GLF is larger than that of LF, and both varieties are diploid. We found that the cell size in GLF fruit was larger than that of LF. Then, we compared the fruit transcriptomes of the two varieties using RNA-Seq technology. A total of 1166 differentially expressed genes (DEGs) were detected between GLF and LF fruits. The KEGG analysis revealed that the phytohormone pathway was the most enriched, in which most of the DEGs were related to auxin signaling. Moreover, the endogenous auxin levels of GLF fruit were higher than those of LF. The expressions of auxin synthetic genes, including *MdTAR1* and *MdYUCCA6*, were higher in GLF fruit than LF. Collectively, our findings suggest that auxin plays an important role in fruit size development.

## Introduction

The apple (*Malus domestica*) is widely cultivated in temperate regions worldwide (Duan et al., 2017; McClure et al., 2018). Fruit size is an important trait that influences the economic value of apple (Zhang et al., 2005; Malladi and Hirst, 2010). Developing an apple variety with a larger fruit size is one of the most important goals for breeders; however, the mechanisms underlying fruit size regulation are poorly understood.

Fruit size is determined by two factors, cell number and/or cell size (Scorzal et al., 1991; Olmstead et al., 2007; Malladi and Hirst, 2010). Previous research has reported that cell number is the major factor influencing fruit size. For example, ectopic expression of *AINTEGUMENTA* (*ANT*), a *APETALA2* (*AP2*)-like domain transcription factor, increased the organ size of *Arabidopsis* by increasing the cell number (Krizek, 1999). Overexpression of *BIG BROTHER*, an E3 ubiquitin ligase gene, reduced the organ size by restricting cell numbers in *Arabidopsis* (Disch et al., 2006). Besides cell number, cell size is also an important factor that controls fruit size. In tomato, the larger fruit varieties have a larger cell size than small fruit varieties because the cell size is positively correlated with fruit size (Cheniclet et al., 2005). In apple, a bud sport variety of “Gala” was identified, named “Grand Gala,” and the fruit of “Grand Gala” is larger than “Gala” due to its larger cell size (Malladi and Hirst, 2010), but the underlying mechanism causing the larger cell size of “Grand Gala” is unclear.

Fruit size is regulated by multiple factors, including phytohormones, and genetic factors. For example, the application of N1-(2-chloro-4-pyridyl)-N3-phenylurea (CPPU), an artificial-synthesized cytokinin, increased fruit size in kiwifruit (*Actinidia chinensis*) by inducing the cell number (Cruz-Castillo et al., 2002). Gibberellin treatment increased the fruit size of pear (*Pyrus pyrifolia*; Ito et al., 2015) and apple (Martin et al., 1970). Auxin has been reported to affect the fruit size in many tree fruits, for example, exogenous auxin treatment increased fruit size by increasing cell size in apple (Devoghalaere et al., 2012).

Auxin is achieved through the coordination of complex processes, including auxin synthesis, metabolism, transport, and signal transduction (Devoghalaere et al., 2012). Indole acetic acid (IAA) is the predominant form of auxin, and the indole-3-pyruvate (IPA) pathway is the predominant path of IAA biosynthesis in plants, which contains two main enzymes, tryptophan aminotransferase of *Arabidopsis*/tryptophan aminotransferase-related (TAA1/TAR), and flavin monooxygenase (YUCCA; Zhao et al., 2001). Additionally, gretchen hagen 3 (GH3) family protein can conjugate amino acids and IAA to form inactive IAA (Staswick et al., 2002). Aside from its synthesis and conjugation, auxin is transported between cells (Zažímalová et al., 2010). Auxin-resistant 1/like auxin-resistant 1 (AUX1/LAX1) mainly transports auxin from extracellular to intracellular regions (Yang et al., 2006; Vanneste and Friml, 2009), and PIN-formed 1 (PIN1) is responsible for auxin transport in the reverse direction (Wabnik et al., 2010). Auxin synthesis, conjugation, and transport are tightly regulated and lead to auxin homeostasis (Perrot-Rechenmann and Napier, 2005).

Changing the concentration of endogenous auxin can modify its signaling response, causing several gene transcription level changes, such as *auxin/indole acetic acid* (*Aux*/*IAA*) and *small auxin up RNA* (*SAUR*; Paponov et al., 2008). When the auxin concentration is low, its signal transduction is blocked by Aux/IAA transcription repressors that interact with auxin response factors (ARFs), thereby repressing their transcription activity (Lavy and Estelle, 2016). When the auxin concentration is elevated, Aux/IAA interacts with the auxin receptor, transport inhibitor response 1/auxin signaling F-BOX protein (TIR1/AFB), which is a component of E3 ubiquitin ligase that undergoes ubiquitin-mediated protein degradation. ARFs are subsequently released, and auxin signaling is activated (Devoghalaere et al., 2012; Leyser, 2018).

Previous studies have elucidated the roles of genes that regulate fruit size. For example, *fruit weight 2.2* (*FW2.2*) is a negative regulator of fruit size and regulates cell number during the early stage of fruit development in tomato (*Solanum lycopersicum*; Frary et al., 2000). In apple, the overexpression of *microRNA172* inhibits the transcription of *AP2*, leading to decreased cell size and significantly reduced fruit size (Yao et al., 2015). Furthermore, the silencing of *MdMADS8* or *MdMADS9* resulted in smaller cell size and greatly reduced fruit size in apple (Ireland et al., 2013).

The “Longfeng” (LF) apple variety is widely cultivated in Northeast China (Li, 1994). Recently, a bud sport variety of LF was identified and named “Grand Longfeng” (GLF). GLF has a larger fruit size than LF; however, it is unclear why GLF apple fruit becomes larger. In this study, we found that the cell size of GLF fruit was larger than LF. The transcriptomes of GLF and LF fruits were also compared and the probable explanation for the larger fruit size of GLF is discussed.

## Materials and Methods

### Plant Materials and Treatment

Longfeng and GLF apple fruits were collected from an orchard (E129°32′12″, N44°18′00″) located in Dongsheng Village, Ningan Town, Mudanjiang City, Heilongjiang Province, China. LF and GLF trees were grown on *M. baccata* rootstocks with normal management. The maturation date of both varieties is around 120 days after full bloom (DAFB). For fruit size measurements, the fruits of both varieties were collected every 21 days (d) from 9 to 120 DAFB and 10 fruits were collected at each sampling point. Fruit core diameter, longitudinal diameter, and transversal diameter were measured with a digital Vernier caliper (PD-151; Pro’skit, Taiwan, China). For the 1-naphthylacetic acid (NAA; BBI Life Sciences, Shanghai, China) treatment, 1 μM NAA was sprayed on LF fruit at 30 DAFB. Fruits treated with distilled water were used as controls. Fruits were harvested at the commercial harvest day (120 DAFB). For the 2,3,5-triiodobenzoic acid (TIBA; Shanghai Maokang Biotechnology Co., Ltd., Shanghai, China) treatment, 100 μM TIBA, which is an inhibitor of auxin polarity transport, was injected into the calyx tube of GLF fruit at 30 DAFB. Fruits injected with distilled water were used as controls. Fruits were harvested at the commercial harvest day (120 DAFB). Transverse and longitudinal diameters were also measured. Fruit weights were measured by electronic scales (JY10002; Sunny Hengping Scientific Instrument Co., Ltd., Shanghai, China). Student’s *t*-test was used for statistical analysis using SPSS v18.0 (IBM, Chicago, Illinois, United States). At each sampling point, the cortex of 10 fruits was sliced, frozen in liquid nitrogen, and stored at the −70°C for future analysis.

### SSR Analysis of GLF and LF Apple Fruit

Genomic DNA was extracted according to previously reported methods (Wang et al., 2013), and 16 pairs of SSR (simple sequence repeat) primers were selected for PCR. Denaturing polyacrylamide gel examining was used for PCR products analysis accord to the method of Li et al. (2020). The primers were listed in Supplementary Table 1.

### Chromosome Ploidy Identification

Fresh leaves were used for ploidy identification using a flow cytometer (FACSCalibar; Beckton Dickinson Co., Franklin lakes, NJ, United States) following the manufacturer’s instructions. About 0.5 cm^2^ of leaf disk was dipped in 400 μL extracting buffer [1% beta-mercaptoethanol, 0.05% Triton X-100, 20 μg mL^–1^ RNase A, 15 mM Tris–HCl (pH 8.0), 2 mM Na_2_EDTA, 20 mM NaCl, and 80 mM KCl], ground into small particles (Zhang et al., 2011), and filtrated through a 500-μm mesh sieve. The filtrate was stained with 20 μg mL^–1^ propidium iodide (Sigma, Louis, Missouri, United States) and incubated in the dark for 15 min at room temperature. After staining, the nuclei were collected by filtering through a 25-μm nylon mesh. Flow cytometry was performed using the flow cytometer. Diploid “Hanfu” apple (*M. domestica*, 2n = 2x = 34) was used as a control and internal reference (Ma et al., 2016). All chemicals were purchased from the TransGen Biotech Co., Ltd. (Beijing, China) unless otherwise indicated.

### Cytological Analysis

Fruit flesh was fixed in FAA (50% ethanol:formaldehyde:glacial acetic acid = 90:5:5) for 24 h, then used for making paraffin sections as previously described (Yao et al., 2015). Sections were cut by a rotary slicer (Leica RM2255; Leica, Wetzlar, Germany) and stained with 1% toluidine blue for 3–5 min. Six consecutive cells were measured between the pericarp and core using a scale tool under a microscope. The average of 6 consecutive cell lengths from the core to the skin was used as the single-cell length. The thickness of the fruit cortex was measured using the digital Vernier caliper. Cell numbers were calculated as the cortex size divided by the single-cell size. Section images were captured using an Olympus BX50f-3 microscope (Olympus Optical Co., Ltd., Tokyo, Japan). Fruits from three trees (one fruit per tree) of each variety were used as 1 biological replicate; a total of three biological replicates were used at each stage. Student’s *t*-test was used for statistical analysis using SPSS v18.0.

### RNA-Sequencing

Longfeng and GLF fruits were collected at 72 DAFB and used for RNA-Seq. Fruits were collected from 3 trees (3 fruits per tree), and the fruit flesh from each tree was equally mixed and used as 1 biological replicate. A total of 3 biological replicates were used. Total RNA was extracted according to previously reported methods (Gambino et al., 2008). cDNA library construction, RNA-Seq, and the bioinformatics analysis were performed by Biomarker Technologies Co., Ltd. (Beijing, China). RNA-Seq was performed using an Illumina HiSeq^TM^ 2500 system (Illumina, San Diego, California, United States).

### Gene Functional Annotation and Enrichment Analysis

Gene functional annotation was performed based on the NCBI non-redundant (Nr) protein sequences, NCBI nucleotide (Nt) sequences, protein family (Pfam), clusters of orthologous groups of proteins (KOG/COG), Swiss-Prot (a manually annotated and reviewed protein sequence database), KEGG ortholog (KO), and gene ontology (GO) databases. The GO enrichment analysis of the differentially expressed genes (DEGs) was implemented using the GOseq R package based on Wallenius non-central hypergeometric distributions (Young et al., 2010). KOBAS software was used to test the statistical enrichment of DEGs in the KEGG pathways (Mao et al., 2005). KEGG annotation of the genes was performed following previously reported methods (Kanehisa et al., 2004).

### Determination of Endogenous IAA Contents

The cortex of the fruit from three trees (three fruits per tree) of each variety was mixed and used as one biological replicate with a total of three biological replicates. Fruit cortex was frozen in liquid nitrogen, ground into a fine powder, and dried under a vacuum (0.08 mbar) at −45°C. Endogenous auxin was measured by gas chromatography–mass spectrometry according to previously described methods with slight modifications (Müller et al., 2002). Ten mg fruit cortex was extracted with MeOH:H_2_O (4:1) as a solvent and using [^13^C_6_] IAA (CLM-1896-0; Cambridge Isotope Laboratories, Inc., Andover, MA, United States) as an internal standard. The extract was evaporated until dry. After briefly cleaning the resuspended dried extract with 80% MeOH (v/v), the fraction containing the phytohormone was collected and dried by a centrifugal concentrator. The IAA in the extracts was trimethylsilylated with N-methyl-N-trimethylsilyl-trifluoroacetamide (MSTFA) at 80°C for 30 min. Samples were freeze dried using vacuum freeze-drying equipment (XYL-LGJ-10D; Beijing Heng Odd Instrument Co., Ltd., Beijing, China) for 24 h and dissolved in hexane before placement in a GC-QqQ MS (7890a-5975b; Agilent, Santa Clara, CA, United States) with a fused silica glass capillary column DB-5 (30 m × 0.25 mm × 0.10 μm; Agilent, Santa Clara, CA, United States). Injection and interface temperatures were 260°C and 280°C, respectively. The column temperature gradient was maintained at 80°C for 2 min, then increased by 6°C min^–1^ to 250°C, followed by 20°C min^–1^ to 300°C. IAA was confirmed by monitoring the diagnostic ions of both endogenous and deuterated hormones according to previously described methods Müller et al. (2002); [^13^C_6_] IAA was used as an internal standard. Student’s *t*-test was used for statistical analysis.

### DEG Analysis

Gene expression levels were determined by fragments per kilobase of transcript per million fragments (FPKM). Differential expression analysis of the sample groups was performed by DESeq (Anders and Huber, 2010). Significant *p*-values were obtained from the original hypothesis test. The false discovery rate (FDR) was obtained using the Benjamini–Hochberg correction method (Anders and Huber, 2010), which was used as a key indicator for DEG screening. The ratio of expression between two sample groups with the screening criteria, fold change >2, and FDR < 0.01 was used to screen the ratio of expression between the two sample groups.

### Quantitative Reverse Transcription-PCR

Longfeng and GLF fruits were collected from three trees (three fruits per tree), and the fruit flesh from each tree was equally mixed and used as one biological replicate. A total of three biological replicates were used. Fruit flesh RNA extraction was performed according to previously described methods (Li et al., 2017). First-strand cDNA was synthesized from 1 μg total RNA using an M-MLV RTase cDNA Synthesis kit (D6130; TaKaRa, Shiga, Japan). Quantitative Reverse Transcription-PCR (qRT-PCR) was performed on a qTOWER3G RT-PCR system (Analytik Jena, Jena, Germany) with a 10-μL total volume containing 5 μL SYBR green master mix (Cat. 04707516001; Roche Diagnostic Ltd, Basel, Switzerland), 0.5 μL cDNA, 0.5 μL reverse and forward primers, and 3.5 μL H_2_O. The reaction programs were performed as follows: 10 min at 95°C, 40 cycles of amplification for 30 s at 95°C, 30 s at 60°C, and 30 s at 72°C, and a final dissociation stage for 6 s at 72°C. Student’s *t*-test was used for statistical analysis. Primer3 software^1^ was used for designing primers. All primer sequences are listed in Supplementary Table 1.

## Results

### GLF Fruits Were Significantly Larger Than LF Fruits

**FIGURE 1:**
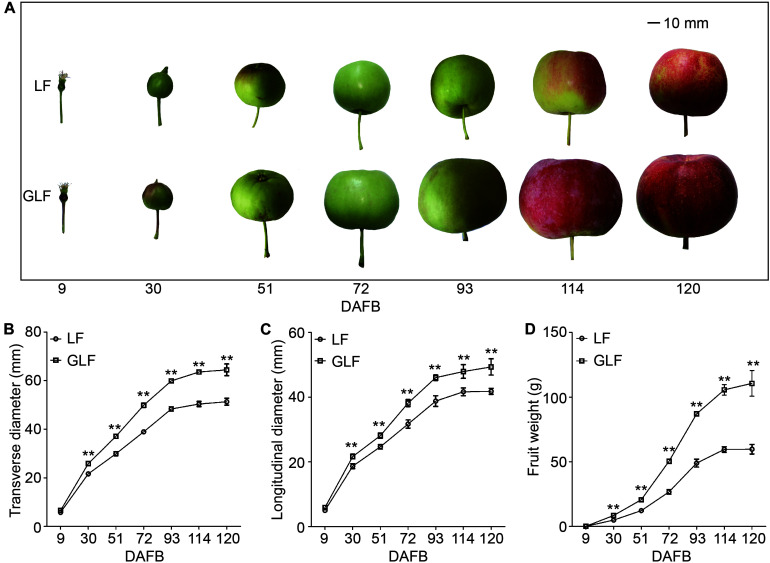
Comparison of LF and GLF fruit growth. LF and GLF fruits were harvested every 21 d from 9 to 120 DAFB (days after full bloom; **A**). Fruit transverse diameter **(B)**, longitudinal diameter **(C)**, and weight **(D)** were measured and compared. **Significant differences (*p* < 0.01, Student’s *t*-test). Error bars indicate the standard deviation (SD) of 10 fruits. Bar, 10 mm.

Grand Longfeng apple is a bud sport variety of LF, which was found in 2003 on a LF tree. GLF showed a fruit size larger than LF (Figure 1A). We then used these two varieties to study the molecular basis for the larger fruit size of GLF. We first compared the genetic background of LF and GLF using 16 pairs of SSR primers (Supplementary Figure 1 and Supplementary Table 1) and observed no difference in SSR band patterns between these two varieties (Supplementary Figure 1), indicating that GLF and LF have high similarity in genetic background. Next, LF and GLF fruit sizes were measured from 9 to 120 DAFB (days after full bloom; Figure 1A). The fruit weight, transverse diameter, and longitudinal diameter of GLF were 1.9, 1.3, and 1.2 × times greater than LF, respectively, at 120 DAFB (Figures 1B–D). No significant differences were detected in the core diameter between the two varieties (Supplementary Figure 2). Thus, it was concluded that the difference in fruit size between GLF and LF was caused by the thickness of the fruit cortex. Then, we examined the ploidy of the two varieties. Results revealed that both were diploid (Supplementary Figure 3).

**FIGURE 2:**
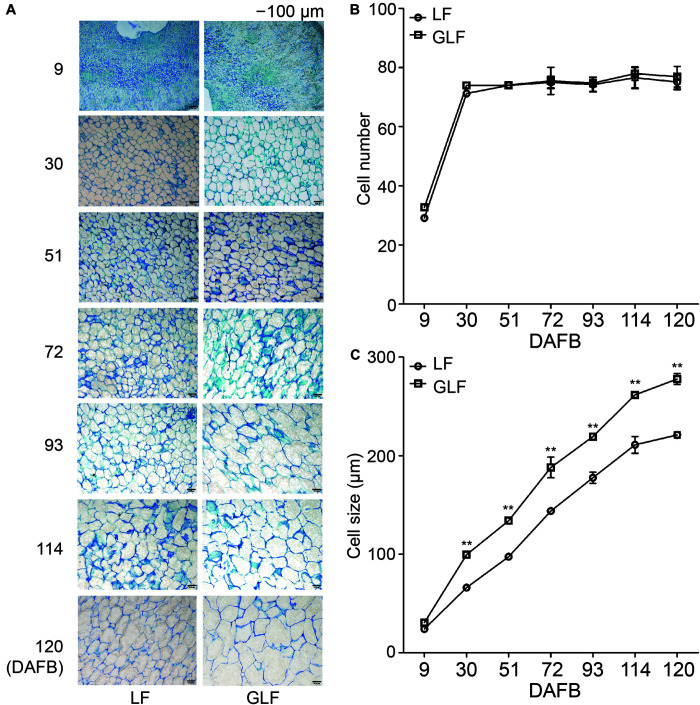
Cell numbers and sizes of LF and GLF fruits. **(A)** Cells of LF and GLF fruits from 9 to 120 DAFB (days after full bloom). Bar, 100 μm. **(B)** Cell numbers of LF and GLF fruits were determined as the ratio between the fruit cortex and single cell size. **(C)** Cell sizes of LF and GLF fruit cortexes were determined as the average diameter of six cells during fruit development. **Significant differences (*p* < 0.01, Student’s *t*-test). Error bars indicate the standard deviation (SD) of three biological replicates.

Next, we compared the cell size and number of the GLF and LF fruit cortexes. The cell size of GLF was significantly larger than that of LF from 30 to 120 DAFB (Figures 2A,C). Interestingly, this timespan coincided with the periods when fruit size differences were detected between the two varieties (Figures 1B–D). Although the cell number of GLF was greater than LF at the early stage (9 DAFB), no significant differences were detected after 30 DAFB (Figure 2B). These results indicated that cell size is a major factor that results in the larger size of GLF fruit.

### Comparison of LF and GLF Fruit Transcriptomes

To identify the cause of the fruit size difference between LF and GLF, we compared the transcriptomes of the two varieties using fruit collected at 72 DAFB (cell enlargement period). Three biological replicates were used and a total of six samples were sequenced. A total of 36.42 GB clean data (6.07 GB clean data for each sample) were obtained. The Q30 percentages of each sample were ≥91.73% (Table 1). The clean reads of each sample were mapped to the apple reference genome^2^ (Daccord et al., 2017). A total of 1166 DEGs were obtained (Supplementary Table 2).

**TABLE 1:** Mapping of RNA-Seq reads obtained from GLF and LF fruits.

Sample	Clean reads (strip)	Clean bases (bp)	GC (%)	Q30 (%)
LF-1	27,537,481	8,221,649,800	47.73	92.67
LF-2	26,912,877	8,043,076,294	47.55	92.37
LF-3	25,693,947	7,665,601,944	47.56	92.38
GLF-1	23,691,600	7,058,675,886	47.49	91.73
GLF-2	24,822,984	7,391,795,358	47.72	92.25
GLF-3	22,064,826	6,589,203,906	47.91	92.55

*LF-1, LF-2, and LF-3 represent three biological replicates of LF at 72 DAFB; GLF-1, GLF-2, and GLF-3 represent three biological replicates of GLF at 72 DAFB. Clean reads (strip) represent the total number of paired-end reads in the clean data; clean bases (bp) represent clean data total base numbers.*

### Functional Annotation of DEGs

All DEGs were aligned by conducting BLASTx searches (*E* values ≤ 10^5^) against the GO, Swiss-Prot, Nr NCBI, KEGG, and COG/KOG protein databases. A total of 1128 DEGs were annotated (Supplementary Table 3). We used the KEGG pathway database to search for functional networks of the biological interactions. A total of 85 KEGG pathways were obtained (Supplementary Table 4). Interestingly, plant hormone signal transduction (ko04075) contained the largest number of genes (38 genes; Supplementary Figure 4), accounting for 16.52% of the ko04075 pathway (230 genes). Additionally, the *Q*-value (where smaller *Q*-values are the most important in terms of DEGs pathway enrichment significance) of the plant hormone signal transduction pathway (*Q* value = 7.82E^–09^) was smaller than all other pathways, indicating that plant hormone signal transduction was the most important pathway (Supplementary Figure 5 and Supplementary Table 4). Among these genes, 30 were associated with the auxin pathway, including six gene families (*AUX1*, *PIN*, *Aux*/*IAA*, *ARF*, *GH3*, and *SAUR*; Supplementary Table 3), suggesting that the auxin pathway considerably contributed to the larger fruit size of GLF.

### qRT-PCR Verification of the DEGs Between GLF and LF Fruits

**FIGURE 3:**
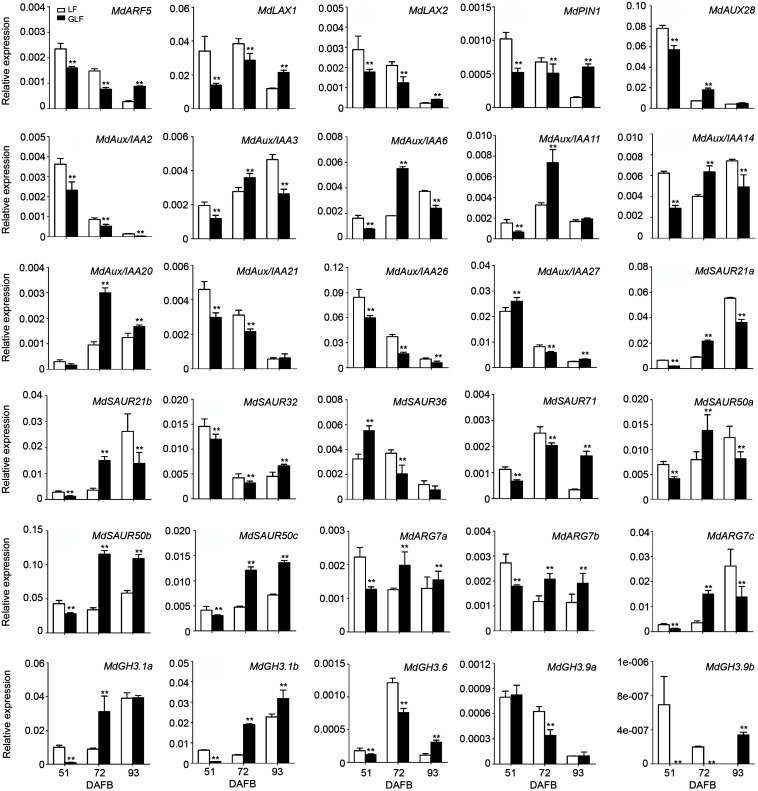
Expression of DEGs in LF and GLF fruits. qRT-PCR was used to measure the expression of DEGs related to auxin signaling in LF and GLF fruits at 51, 72, and 93 DAFB (days after full bloom). **Significant differences (*p* < 0.01, Student’s *t*-test). Error bars indicate the standard deviation (SD) of three biological replicates.

To confirm the accuracy of the transcriptome data, 30 of auxin signaling genes were selected for qRT-PCR comparison between GLF and LF fruits. Results revealed a positive correlation with the RNA-Seq data in 72 DAFB samples (Figure 3 and Supplementary Table 3), which confirmed the accuracy of the transcriptome results.

**FIGURE 4:**
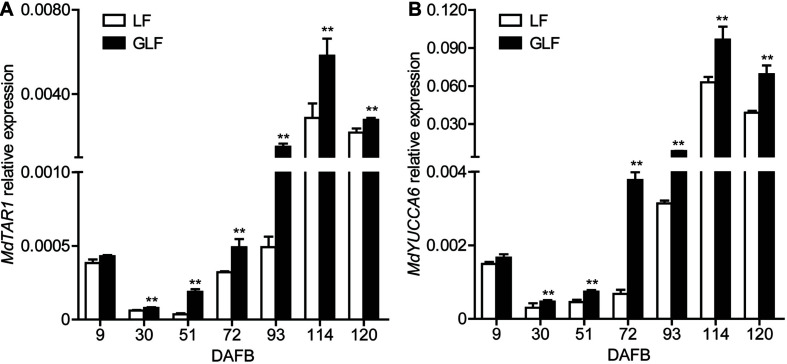
Relative expression of *MdTAR1* and *MdYUCCA6* in LF and GLF fruits. GLF and LF fruit cortexes were collected from 9 to 120 DAFB (days after full bloom). qRT-PCR was used to measure the relative expression of *MdTAR1* **(A)** and *MdYUCCA6* **(B)**. **Significant differences (*p* < 0.01, Student’s *t*-test). Error bars indicate the standard deviation (SD) of three biological replicates.

Auxin synthesis, conjugation, and transport are tightly regulated, leading to auxin homeostasis (Perrot-Rechenmann and Napier, 2005). Changing endogenous auxin concentrations can modify the signaling response, causing several gene transcription level changes (Paponov et al., 2008). Combined with the transcriptome results, two genes (*MdTAR1* and *MdYUCCA6*) were found to be responsible for auxin synthesis and upregulated in GLF at 72 DAFB (Supplementary Table 3). Thus, we proposed that the upregulation of auxin synthetic genes may lead to increased auxin concentrations. Then, we investigated the transcription levels of *MdTAR1* and *MdYUCCA6* by qRT-PCR. *MdTAR1* and *MdYUCCA6* were expressed at higher levels in GLF than LF at six fruit development stages (30, 51, 72, 93, 114, and 120 DAFB; Figure 4), suggesting that the upregulation of these auxin synthetic genes may lead to differential auxin levels, thereby leading to larger fruit sizes in GLF.

### Endogenous Auxin Levels Were Higher in GLF Than LF Fruit

**FIGURE 5:**
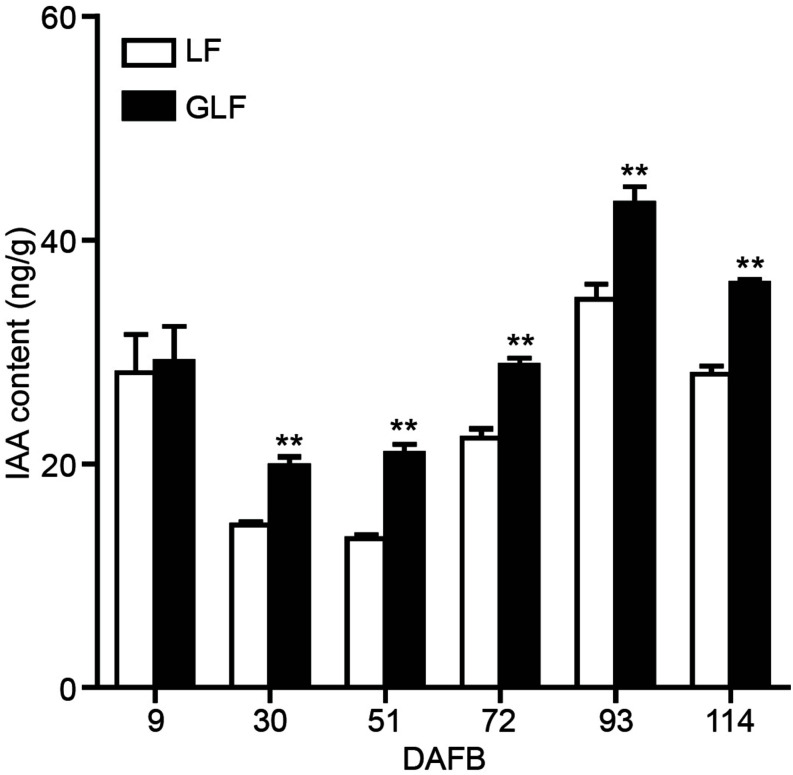
Auxin contents of LF and GLF fruits. The IAA content was measured using fruit cortex of LF and GLF. **Significant differences (*p* < 0.01, Student’s *t*-test). Error bars indicate the standard deviation (SD) of three biological replicates.

**FIGURE 6:**
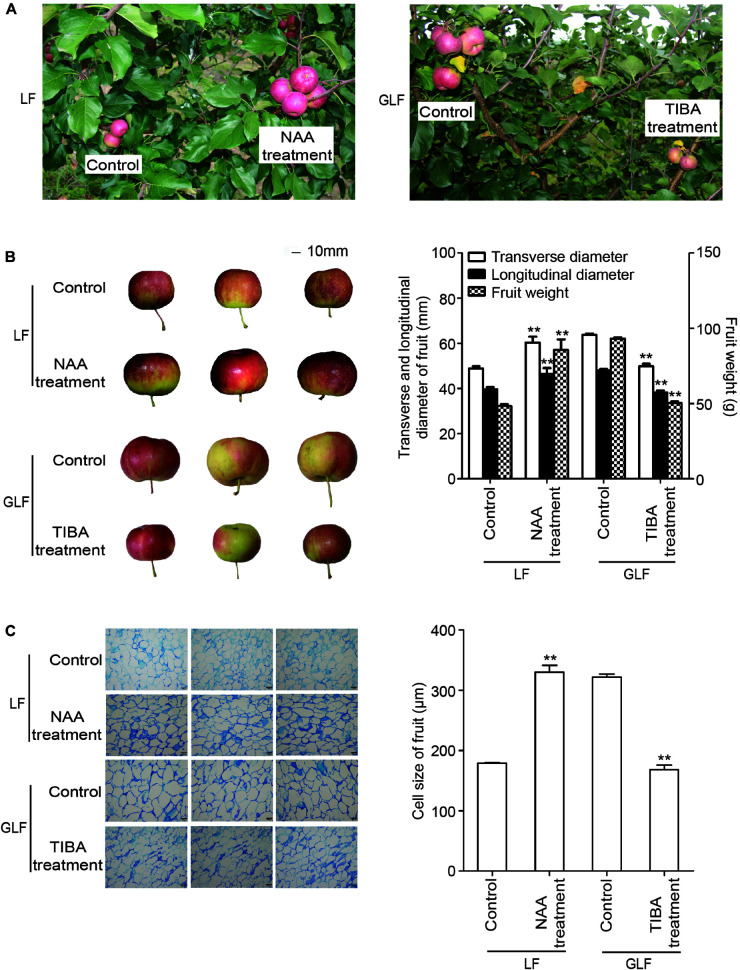
The influence of auxin and TIBA on fruit and cell sizes. On-tree LF and GLF fruits were treated with NAA and TIBA at 30 DAFB (days after full bloom), respectively, and harvested at 120 DAFB **(A)**. Fruit transverse diameter, longitudinal diameter, and weight were measured on harvest fruit. Bar, 10 mm **(B)**. Cell sizes were calculated as the average of six typical cell lengths, which were measured by a scale tool under a microscope at 120 DAFB. Bar, 100 μm **(C)**. **Significant differences (*p* < 0.01, Student’s *t*-test). Error bars indicate the standard deviation (SD) of three biological replicates.

Since the expressions of two auxin synthetic genes (*MdTAR1* and *MdYUCCA6*) were higher in GLF fruit than in LF fruit, we speculated that upregulation of auxin synthetic genes may lead to increased auxin in GLF. The endogenous IAA levels of both varieties were measured. Results revealed that the endogenous IAA content in GLF was significantly higher than that in LF at 30, 51, 72, 93, and 114 DAFB (Figure 5). To determine whether auxin levels affected fruit size, NAA was used to treat the on-tree fruit of LF at 30 DAFB. Interestingly, the NAA treatment significantly increased the fruit weight, transverse diameter, and longitudinal diameter of LF when harvested at 120 DAFB (Figures 6A,B). In addition, LF cell sizes were significantly enlarged after NAA treatment (Figure 6C). Next, we used TIBA, an inhibitor of auxin transport polarity, to investigate the effects of the auxin reduction on fruit size. The TIBA treatment significantly decreased the fruit weight, transverse diameter, and longitudinal diameter of GLF when harvested at 120 DAFB (Figures 6A,B). GLF cell sizes were significantly reduced by the TIBA treatment (Figure 6C). These results suggested that the higher levels of endogenous IAA may result in the larger fruit size of GLF.

## Discussion

Fruit size is an important trait that influences the economic value of fruit (Malladi and Hirst, 2010). Multiple factors influence fruit size, including ploidy, hormone levels, and genetic controls. For example, the tetraploid “Hanfu” apple has a larger fruit size than the diploid “Hanfu” apple (Xue et al., 2017). In this study, we found that both GLF and LF were diploid (Supplementary Figure 3); thus, ploidy could not explain the larger fruit size of GLF. Previous studies have revealed that cell size and number play important roles in affecting the size of different fruit, including tomato (Cheniclet et al., 2005), sweet cherry (Olmstead et al., 2007), and peach (Guo et al., 2018). Here, we found that the large fruit size of GLF correlated with cell size (Figure 2C). This result supports the findings of Malladi and Hirst (2010), in which cell size was a major factor affecting the larger size of “Grand Gala” fruit, a bud sport variety of “Gala” apple. In the early stage of fruit development, it involves both cell division and increase in cell size or cell growth due to addition of additional cellular contents. At later stages, increase in cell size is greatly aided by post-mitotic cell expansion which would also involve greater vacuolation (Warrington et al., 1999; Janssen et al., 2008). In our data, the cell numbers increased greatly from 9 to 30 DAFB and remained the same at later stages (Figure 2B), but the cell size was much greater in GLF than in LF throughout fruit development (Figure 2). Thus, the cell growth that enhanced during early fruit development of GLF might continue at later stages, leading to larger fruit of GLF. Based upon this, we selected samples of 72 DAFB for RNA-seq.

Previous studies have elucidated the roles of various genes involved in fruit size. For example, *ANT* or *BIG BROTHER* regulated organ size by controlling cell numbers in *Arabidopsis* (Krizek, 1999; Disch et al., 2006). *WEE1* regulated fruit size by controlling cell size in tomato fruit (Gonzalez et al., 2007), and *FW2.2* negatively regulated cell proliferation, which thereby influenced the fruit size (Frary et al., 2000). Silencing of *PaCYP78A9* reduced fruit size through its effect on reducing cell size and cell number in sweet cherry (Qi et al., 2017). In apple, *microRNA172* overexpression inhibits the transcription of *AP2*, conferring significantly reduced fruit size (Yao et al., 2015). Suppression of *MdMADS8* or *MdMADS9* expression significantly reduced cell size, resulting in smaller apple fruits (Ireland et al., 2013). In this study, these genes did not exhibit differential expression between GLF and LF, based on the RNA-Seq data (Supplementary Table 3). In addition, fruit size is regulated by phytohormones. For example, the application of CPPU, an artificial-synthesized cytokinin, increased fruit size in kiwifruit (*A. chinensis*) by inducing cell number (Cruz-Castillo et al., 2002). Gibberellin treatment increased the fruit size of pear (*P. pyrifolia*; Ito et al., 2015) and apple (Martin et al., 1970). However, in this study, we did not find DEGs belong to cytokinin or gibberellin synthesis genes (Supplementary Table 3). These results suggested the different mechanisms for the formation of larger fruit size of GLF apple.

It was previously reported that auxin increased fruit size by increasing cell size in “Royal Gala” apple (Devoghalaere et al., 2012). In this study, the RNA-Seq analysis revealed 32 DEGs involved in the auxin pathway (Supplementary Table 3), among which 2 auxin synthesis genes (*MdTAR1* and *MdYUCCA6*) were upregulated in GLF (Figure 4). Induction of *TAR* expression increased the IAA concentrations in grapevine (*Vitis vinifera*; Bottcher et al., 2013); Moreover, when *YUCCA6* was overexpressed in the *Arabidopsis yuc6-1D* mutant, it increased the free IAA levels and displayed typical high-auxin phenotypes (Kim et al., 2011). In this study, the endogenous auxin levels were higher in GLF than LF (Figure 5), and the NAA treatment increased the fruit and cell size of LF, while the TIBA treatment decreased the fruit and cell size of LF (Figure 6). Thus, we proposed that the upregulation of these auxin synthetic genes may lead to increased endogenous auxin levels in GLF, resulting in larger cell and fruit sizes than LF. In the future, it will be interesting to investigate what underlying factors determine the differential expression of *MdTAR1* or *MdYUCCA6* between GLF and LF fruits, which may help explain the larger fruit size of GLF.

## Data Availability Statement

Sequence data from this article can be found in the Genome Database for Rosaceae (https://www.rosaceae.org/) or GenBank/EMBL libraries under the following accession numbers: *MD15G1014400 (MdARF5), MD07G1215900 (Md LAX1), MD12G1162400 (MdLAX2), MD06G1226800 (MdPIN1), MD10G1192900 (MdAUX28), MD08G1111200 (MdAux/IAA2), MD04G1225000 (MdAux/IAA3), MD17G1198100 (MdAux/IAA6), MD10G1176400 (MdAux/IAA11), MD13G1205000 (MdAux/IAA14), MD15G1169100 (MdAux/IAA20), MD09G 1208000 (MdAux/IAA21), MD09G1202300 (MdAux/IAA26), MD15G1191800 (MdAux/IAA27), MD10G1059800 (MdSA UR21a), MD10G1059700 (MdSAUR21b), MD16G1124300 (MdSAUR32), MD02G1205700 (MdSAUR36), MD05G1223400 (MdSAUR71), MD05G1052100 (MdSAUR50d), MD10G1059200 (MdSAUR50b), MD10G1059600 (MdSAUR50c), MD15G1222900 (MdARG7a), MD02G1100000 (MdARG7b), MD05G1052400 (MdARG7c), MD05G1092300 (MdGH3.1a), MD05G1092900 (MdGH3.1b), MD11G1230400 (MdGH3.6), MD11G1304200 (MdGH3.9a), MD03G1284700 (MdGH3.9b), MD16G1098400 (MdTAR1)*, and *MD15G1098700 (MdYUCCA6)*. The raw RNA-Seq data were deposited to the NCBI sequence read archive (SRA) database under the accession number, PRJNA551702.

## Author Contributions

HB and AW conceived this project and designed the work. HB, YW, and PY performed the research. WY and HY analyzed the data. HB and AW wrote the manuscript. All authors contributed critically to the drafts and gave final approval for publication.

## Conflict of Interest

The authors declare that the research was conducted in the absence of any commercial or financial relationships that could be construed as a potential conflict of interest.

## Footnotes

1 http://frodo.wi.mit.edu/

2 https://iris.angers.inra.fr/gddh13/

## Supplementary Material

Supplementary Figure 1 Simple sequence repeat (SSR) analysis of LF and GLF. Genomic DNA was isolated from LF and GLF fruits, and 16 pairs of SSR primers were selected for PCR. Denaturing polyacrylamide gel examining was used for PCR products analysis, primer names were indicated using the numbers under the figure. L: LF; G: GLF; and M, DNA size marker.

Supplementary Figure 2 Comparison of core diameter and seed number between LF and GLF fruits. LF and GLF fruits were harvested at 120 DAFB and used for the comparison between core diameter and seed number. Error bars indicate the standard deviation (SD) of 10 fruits.

Supplementary Figure 3 Ploidy analysis of LF and GLF fruits. Young leaves were used to analyze the ploidy of LF **(B)** and GLF **(C)** fruit by flow cytometry. “Hanfu” apple (*M. domestica*, 2n = 2x = 34; **A**), which is diploid, was used as the control and internal reference in **(D,E)**.

Supplementary Figure 4 KEGG analysis of the DEGs between GLF and LF fruits.

Supplementary Figure 5 KEGG enrichment analysis of the DEGs between GLF and LF fruits. The top 20 enriched pathways of the DEGs in GLF fruit were compared to LF. The *x*-axis represents the rich factor on a scale from 2 to 5. The color of the circles represents the *Q*-value. The size of the circles represents the gene number (shown on the right).

Supplementary Table 1 List of primers used in this study.

Supplementary Table 2 DEGs between GLF and LF fruits.

Supplementary Table 3 Annotation of DEGs between GLF and LF fruits.

Supplementary Table 4 KEGG classifications of assembly and enrichment analysis of all DEGs.
