# Supplementary figures and images for: Endogenous Auxin Content Contributes to Larger Size of Apple Fruit
Source: Front Plant Sci. 2020 Dec 3;11:592540. doi: 10.3389/fpls.2020.592540 (PMC7841441; doi:10.3389/fpls.2020.592540)

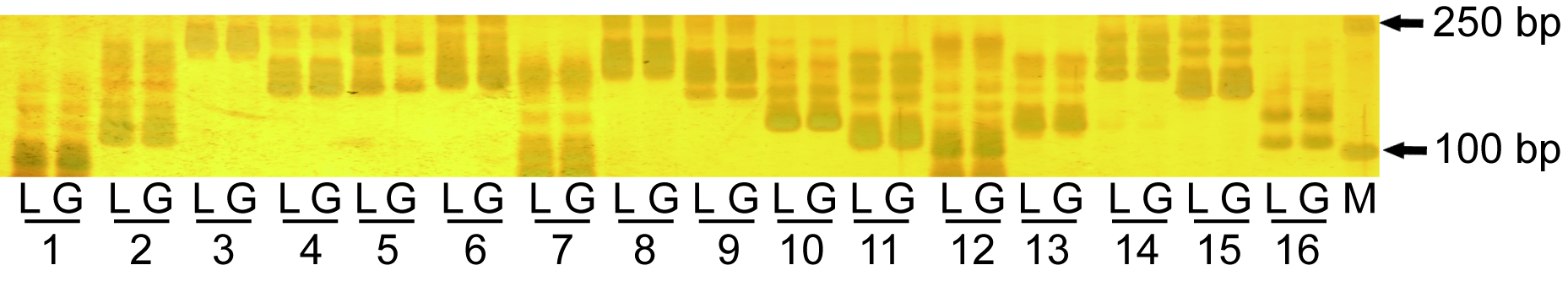

Supplement: Supplementary Figure 1 — Simple sequence repeat (SSR) analysis of LF and GLF. Genomic DNA was isolated from LF and GLF fruits, and 16 pairs of SSR primers were selected for PCR. Denaturing polyacrylamide gel examining was used for PCR products analysis, primer names were indicated using the numbers under the figure. L: LF; G: GLF; and M, DNA size marker. [file Data_Sheet_1.ZIP › Figure S1. Simple sequence repeat (SSR) results of LF and GLF..tif]

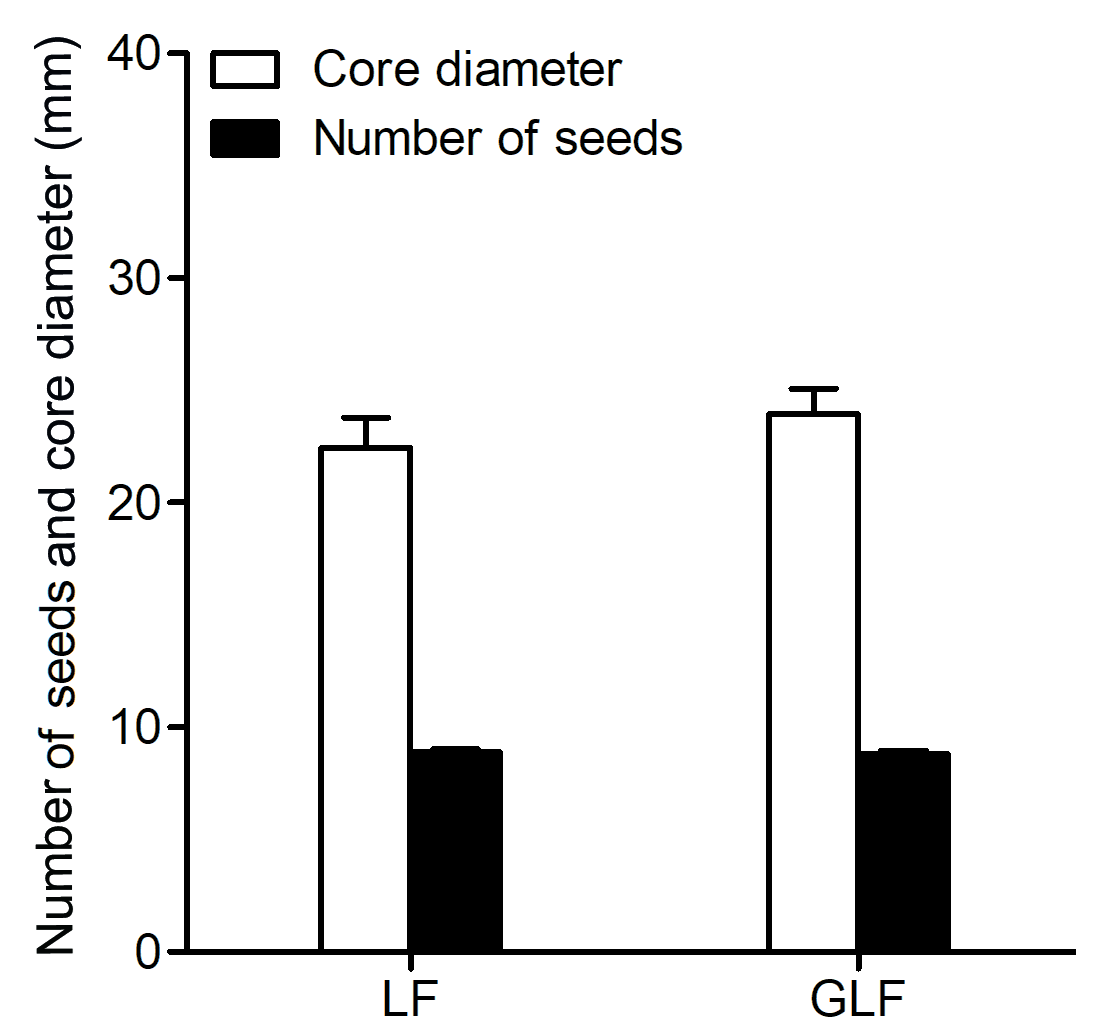

Supplement: Supplementary Figure 1 — Simple sequence repeat (SSR) analysis of LF and GLF. Genomic DNA was isolated from LF and GLF fruits, and 16 pairs of SSR primers were selected for PCR. Denaturing polyacrylamide gel examining was used for PCR products analysis, primer names were indicated using the numbers under the figure. L: LF; G: GLF; and M, DNA size marker. [file Data_Sheet_1.ZIP › Figure S2. Comparison of core diameter and seed number between LF and GLF fruits..tif]

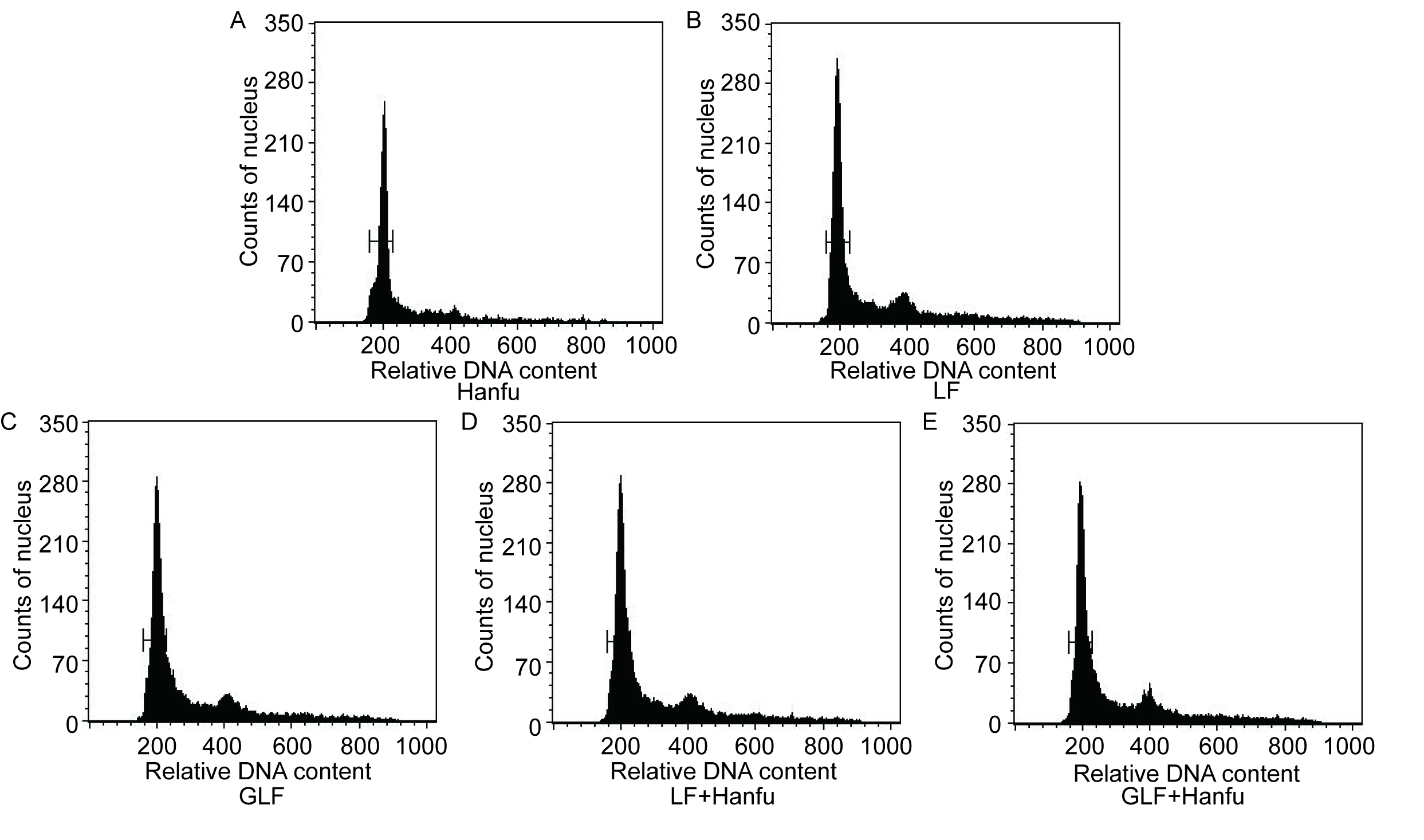

Supplement: Supplementary Figure 1 — Simple sequence repeat (SSR) analysis of LF and GLF. Genomic DNA was isolated from LF and GLF fruits, and 16 pairs of SSR primers were selected for PCR. Denaturing polyacrylamide gel examining was used for PCR products analysis, primer names were indicated using the numbers under the figure. L: LF; G: GLF; and M, DNA size marker. [file Data_Sheet_1.ZIP › Figure S3. Ploidy analysis of LF and GLF fruits..tif]

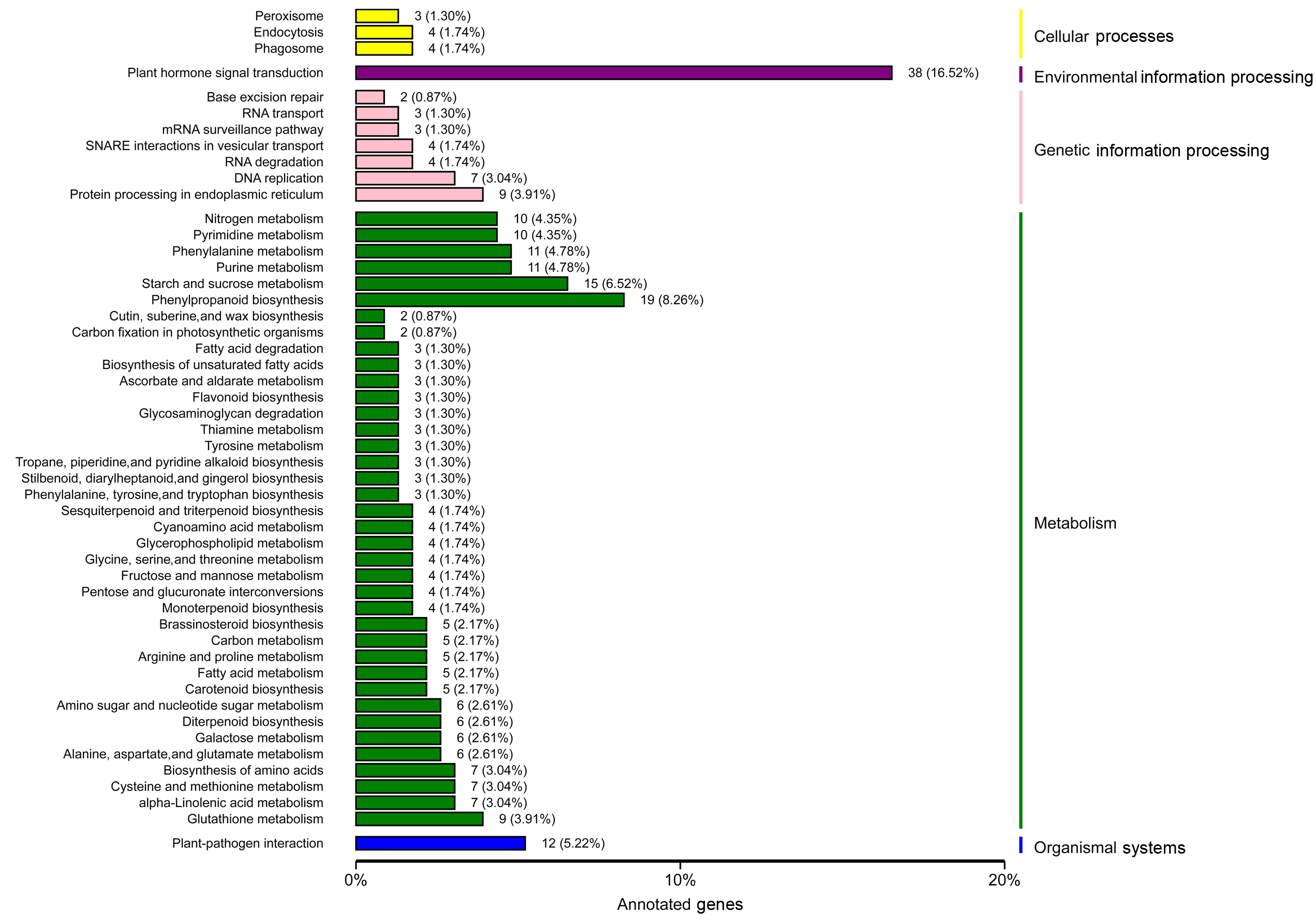

Supplement: Supplementary Figure 1 — Simple sequence repeat (SSR) analysis of LF and GLF. Genomic DNA was isolated from LF and GLF fruits, and 16 pairs of SSR primers were selected for PCR. Denaturing polyacrylamide gel examining was used for PCR products analysis, primer names were indicated using the numbers under the figure. L: LF; G: GLF; and M, DNA size marker. [file Data_Sheet_1.ZIP › Figure S4. KEGG analysis of the DEGs between GLF and LF fruits..tif]

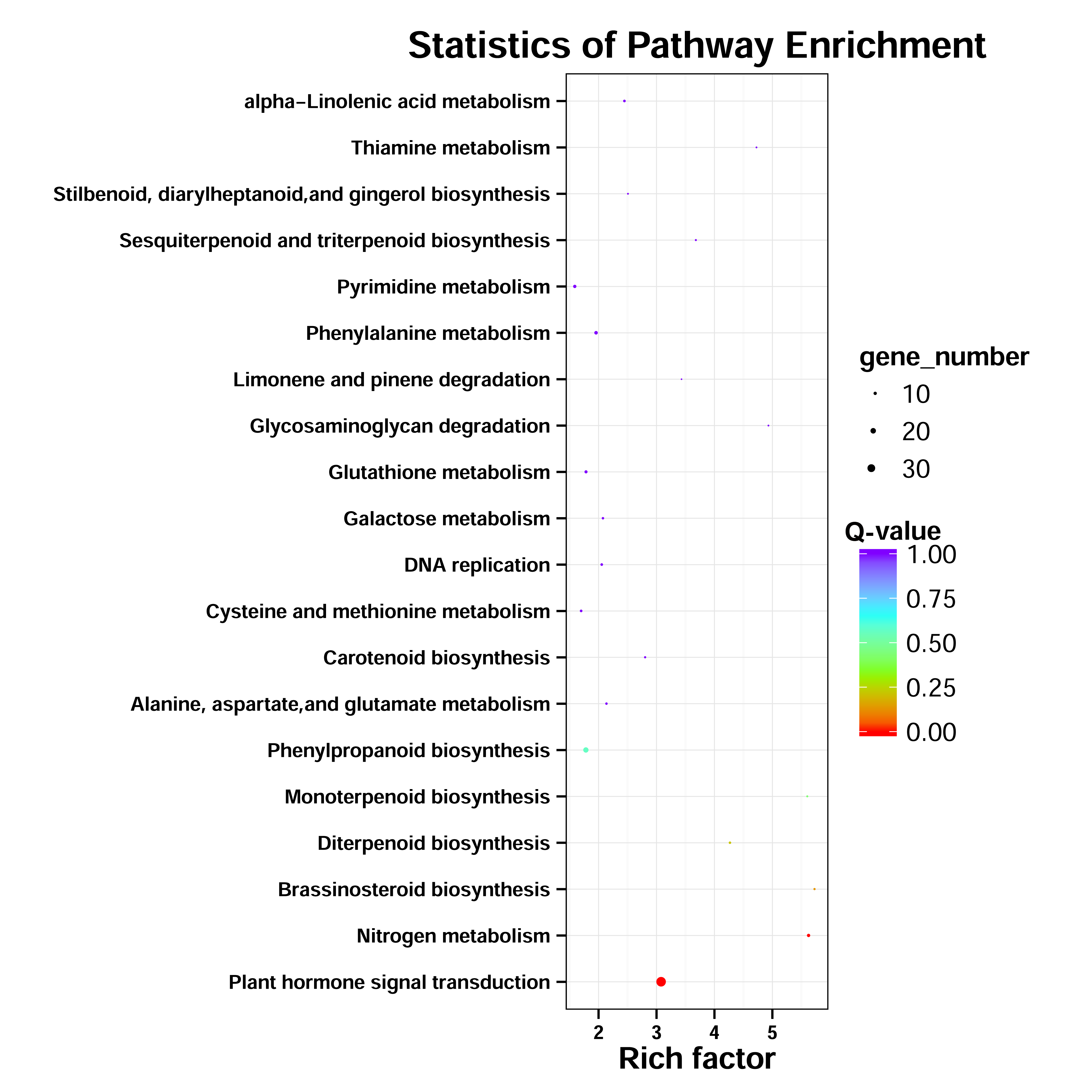

Supplement: Supplementary Figure 1 — Simple sequence repeat (SSR) analysis of LF and GLF. Genomic DNA was isolated from LF and GLF fruits, and 16 pairs of SSR primers were selected for PCR. Denaturing polyacrylamide gel examining was used for PCR products analysis, primer names were indicated using the numbers under the figure. L: LF; G: GLF; and M, DNA size marker. [file Data_Sheet_1.ZIP › Figure S5. KEGG enrichment analysis of the DEGs between GLF and LF fruits..tif]
